# Dynamic strain and β-catenin mediated suppression of interferon responsive genes in quiescent mesenchymal stromal/stem cells

**DOI:** 10.1016/j.bbrep.2024.101847

**Published:** 2024-10-23

**Authors:** Parisa Dashti, Eric A. Lewallen, Gary S. Stein, Bram C.J. van der Eerden, Johannes P.T.M. van Leeuwen, Andre J. van Wijnen

**Affiliations:** a Department of Internal Medicine, Erasmus MC, Erasmus University Medical Center, Rotterdam, Netherlands; b Department of Orthopedic Surgery, Mayo Clinic, Rochester, MN, USA; c Department of Biological Sciences, Hampton University, Hampton, VA, USA; d Department of Biochemistry, University of Vermont, Burlington, VT, USA

**Keywords:** Dynamic strain, Mesenchymal stem cell, Osteoblast, Bone, Chromatin, Epigenetics

## Abstract

Multipotent bone marrow mesenchymal stromal/stem cells (MSCs) respond to mechanical forces. MSCs perceive static and dynamic forces through focal adhesions, as well as cytoskeletal and intranuclear actin. Dynamic strain stimulates nuclear β-catenin (Ctnnb1) that controls gene expression and suppresses osteogenesis. The sensitivity of MSCs to external mechanical forces may be altered by cessation of proliferation, when MSCs begin to express extracellular matrix (ECM) proteins and generate cell/cell contact. Therefore, we assessed whether and how gene expression of proliferating versus quiescent MSCs responds to mechanical stimuli. We used RNA-seq and RT-qPCR to evaluate transcriptomes at 3 h after dynamic strain (200 cycles × 2 % for 20 min) once daily during a two-day time course in naïve (uninduced) MSCs. Transcriptomes of untreated MSCs show that cells become quiescent at day 2 when proliferation markers are downregulated, and ECM related genes are upregulated. On both day 1 and day 2, dynamic strain stimulates expression of oxidative stress related genes (e.g., Nqo1, Prl2c2, Prl2c3). Strikingly, in quiescent MSCs, we observe that dynamic strain suppresses multiple interferon (IFN) responsive genes (e.g., Irf7, Oasl2 and Isg15). IFN responsive genes are activated in MSCs depleted of β-catenin using siRNAs, indicating that β-catenin normally suppresses these genes. Our data indicate that the functional effects of dynamic strain and β-catenin on IFN responsive genes in MSCs are mechanistically coupled. Because dynamic strain and β-catenin reduce the osteogenic potential of MSCs, our findings suggest that IFN responsive genes are novel biomarkers and potential regulators of mechanical responses at early stages of lineage-commitment in post-proliferative MSCs.

## Highlights

• Dynamic strain modulates differentiation of mesenchymal stromal/stem cells (MSCs).
• Transcriptomes of MSCs primarily respond to dynamic strain in a post-proliferative state.
• Dynamic strain downregulates interferon (IFN) responsive genes in quiescent MSCs.
• Loss of β-catenin activates IFN responsive genes in confluent MSCs.
• IFN responsive genes may control early lineage-commitment of MSCs.

## Graphical abstract

**Figure:**
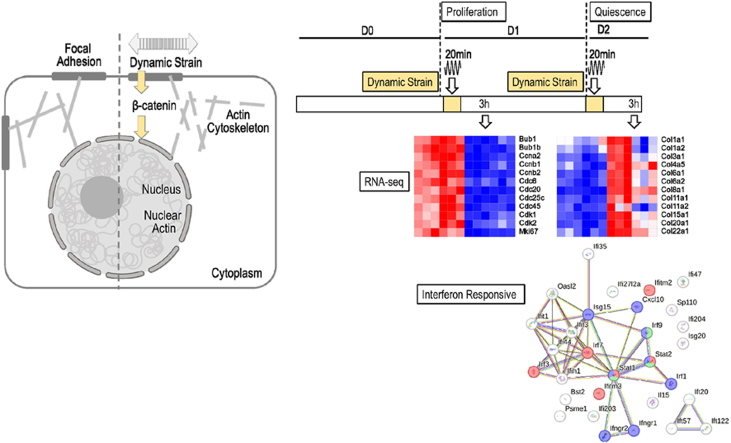

## 1 Introduction

Osteogenesis is controlled by both soluble factors and architectural parameters that orchestrate mesenchymal lineage commitment. These processes generate a pool of multipotent mesenchymal stromal stem cells (MSCs) and skeletal stem cells (SSCs). These stem cell types can differentiate into either osteoblasts required for skeletal remodeling or mature adipocytes for energy storage. Osteogenic lineage commitment and bone formation is regulated by cell signaling, transcription factors and epigenetic regulators controlling chromatin structure in the nucleus [1]. In addition, mechanical forces modulate differentiation of mesenchymal stromal/stem cells (MSCs) [2] that have multilineage potential in culture [3]. The phenotype of MSCs depends in part on the stiffness of the extracellular microenvironment [4,5] that generates tension in cytoarchitecture (i.e., actin and tubulin fibers) to generate tensional integrity (tensegrity) [6]. Increased stiffness of the extracellular matrix influences the nuclear lamina (via lamin A) and supports osteogenesis [5]. Basal static forces due to intrinsic tension between MSCs and their extracellular matrix provides a homeostatic ground state that can be modulated by altering the stiffness of the substrate and alter osteogenic lineage commitment [5].

Extrinsic mechanical stimuli at the cell level modulate gene expression through effects on cytoskeletal and nuclear actin that control chromatin organization [2,[7], [8], [9], [10], [11], [12], [13]]. Mechanical forces [14,15] signal through focal adhesions that anchor the F-actin cytoskeleton to the extracellular matrix [16] and transmit forces through actin polymers directly linked to the nucleus [14,17,18]. Mechanotransduction generates cellular awareness of a dynamic mechanical load and creates a molecular memory in chromatin [19,20]. Chemical perturbation of actin cyto-architecture using inhibitors of actin polymerization (e.g., cytochalasin D) promotes osteogenesis [[21], [22], [23]]. While static strain supports osteogenic differentiation by promoting Hippo/YAP1 signaling in the nucleus, nuclear import of the WNT responsive β-catenin protein reduces osteogenesis and promotes adipogenic differentiation [2,10,[24], [25], [26], [27]]. One exception to this concept comes from studies with canine kidney cells, where proteins moved to the nucleus upon exposure to a high-magnitude static stretch, with YAP1 moving quickly and β-catenin taking over 6 h [28]. The transport of YAP1 and β-catenin to the nucleus proceeds via nuclear pores to permit interactions of these proteins with their respective target genes.

Mechanical effects at the cellular level have intricate and incompletely understood biological relationships with macroscopic forces that support mechanical loading of the skeleton during exercise [29]. For example, macroscopic mechanical loading of bone increases bone mass, while dynamic strain of cultured MSCs reduces osteogenic differentiation [29]. Mechanical signals impact the structure and functionality of bone marrow microenvironment, while MSCs react to both static forces from their location on mineralized tissue and dynamic forces from exercises and other physical activity [30]. Exercise-induced dynamic forces promote bone formation [24,31,32] indicating that dynamic stimulation favors osteogenic over adipogenic differentiation of MSCs *in vivo*. High levels of physical exercise cause considerable deformation of MSCs and bone surfaces to promote osteogenesis. MSCs may differentiate into fat cells in the absence of dynamic pressures [26,32]. Unloading of bone (e.g. due to long term immobilization, cachexia) as well as conditions such as aging and caloric restriction (e.g., anorexia) may change MSC cell fate allocation, resulting in the production of more adipocytes and fewer osteoblasts [[33], [34], [35], [36]]. During bone repair, stromal cell migration or variations in matrix stiffness may result in tissue-specific basal static forces [5] as cells occupy different microenvironments. Bone and other tissues represent different biological contexts that integrate several distinct biomechanical signal types that differ in frequency and magnitude [37]. The development of precise exercise protocols may favor the destiny of MSCs towards osteogenesis and would be a potential strategy for treating low bone mass and bone fragility.

The actin-dependent nuclear entry of β-catenin upon application of dynamic strain preserves the self-renewing undifferentiated state of MSCs [27,28,38]. Stimulation of nuclear β-catenin results in stimulation of EZH2 protein levels [27], while perturbation of actin polymerization results in the downregulation of EZH2 to increase osteogenesis [22,23]. EZH2 is an epigenetic master regulator that modulates skeletal development and MSC differentiation [[39], [40], [41], [42], [43], [44], [45]] in association with a network of other epigenetic enzymes and downstream signaling pathways, including SMYD2 [46], PRMT proteins [47] and GPRC5C [48]. Inhibition of EZH2 results in the reorganization of chromatin to activate key transcription factors (e.g., RUNX2 and SP7) and the osteogenic differentiation program. Importantly, dynamic loading of MSCs increases EZH2 activity [48], which is known to generate an epigenetic regulatory barrier for osteogenic differentiation [1].

Previous transcriptome analysis revealed that MSCs switch from a proliferative mode to a quiescent state, while switching from expression of primarily cytoskeletal genes (e.g., tubulin) to expression of extracellular matrix proteins (e.g., collagens types 1 and III, fibronectin, decorin) [49]. Hence, quiescent MSCs create an external scaffold that may shield the internal cytoskeleton from mechanical forces. Furthermore, quiescence often coincides with cell/cell contact that prevents further proliferation and may result in stress shielding. Therefore, it is important to assess whether MSCs respond differently to mechanical stimuli in proliferative versus quiescent states. Beyond the basic tension in MSCs induced by substrate interactions, cells can be subjected to additional external mechanical modulations. For example, external mechanical forces can be applied by a persistent stretching of cells for some period (‘static strain’), while such forces can also be applied by a cyclical stretching of cells with some frequency during the same time period (‘dynamic strain’). We used RNA-seq and RT-qPCR to show that dynamic strain and β-catenin modulate expression of IFN responsive genes, suggesting that these proteins mediate responses to dynamic mechanical forces in post-proliferative MSCs.

## 2 Materials and methods

### 2.1 MSC isolation and application of mechanical forces

Bone-marrow-derived MSCs from 8 to 10-week-old male C57BL/6 mice were isolated, as described in Ref. [50], from multiple mouse donors and MSC pools, providing a heterogenous MSC cell population. Briefly, tibial and femoral marrow was collected in RPMI1640, 9 % FBS, 9 % HS, 100 μg/mL of penicillin/streptomycin, and 12 μM l-glutamine. After 24 h, non-adherent cells were removed by washing with phosphate-buffered saline and adherent cells cultured for 4 weeks. Passage 1 cells were collected after incubation with 0.25 % trypsin/1 mM EDTA × 2 min, and re-plated in a single 175 cm2 flask. After 1–2 weeks, passage 2 cells were re-plated at 50 cells/cm2 in expansion medium (Iscove modified Dulbecco's, 9 % FBS, 9 % HS, antibiotics, l-glutamine). MSCs were re-plated every 1–2 weeks for two consecutive passages up to passage 5 and tested for osteogenic and adipogenic potential and subsequently frozen.

Uniform biaxial strain was applied to MSCs plated at 10,000 cells per well on 6-well Bioflex Collagen-I-coated plates using the Flexcell FX6000T system (Flexcell International, Hillsborough, NC). The dynamic mechanical strain conditions were previously shown to activate β-catenin [14,27,51] and consisted of subjecting cells to 2 % dynamic strain at 10 cycles per minute for 20 min (200 cycles) at one day (day 1) and two days (day 2) after plating. Control cells were incubated under identical conditions without strain on either day.

### 2.2 High throughput RNA sequencing

RNA sequencing results were obtained and processed as described in detail previously [49,52,53]. RNA samples were indexed using TruSeq Kits and subjected to paired-end sequencing (HiSeq 2000 sequencer) with quality control of the indexed libraries (i.e., Bioanalyzer DNA 1000 chip, Agilent; and Qubit fluorometry, Invitrogen). Read alignments and normalized gene counts expressed as fragments per kilobasepair per million mapped reads (FPKM) were established with the MAP-RSeq (v.1.2.1) pipeline with TopHat and HTSeq, and analyzed for differentially expressed genes using DESeq2, PCA analysis and volcano plots as described previously [46,49,[52], [53], [54]]. Bioinformatic data for specific genes were visualized using heatmaps with log2(x+1) adjustment of expression values and row clustering based on one minus Pearson correlation (Morpheus, https://software.broadinstitute.org/morpheus). Genes were annotated using DAVID6.8 [52] and protein/protein networks were generated using STRING [55].

### 2.3 Quantitative real-time reverse transcriptase PCR (RT-qPCR)

Cells were lysed using TRIzol Reagent (Invitrogen) and RNA was isolated using the Direct-zol RNA MiniPrep Kit (Zymo Research) with an on-column DNA digestion kit (Zymo Research). Isolated RNA was reverse transcribed into cDNA using the SuperScript III First Strand Synthesis System (Invitrogen). Gene expression was quantified using real-time PCR in which each reaction was performed with 10 ng cDNA per 10 μl, QuantiTect SYBR Green PCR Kit (Qiagen), and the CFX384 Real-Time System machine (BioRad). Transcript levels were quantified using the 2 -ΔΔCt method and normalized to the housekeeping gene Gapdh. We used the following gene specific primers:

Nqo1: F- GCCGAACACAAGAAGCTGGAAG, R-GGCAAATCCTGCTACGAGCACT;

Prl2c2: F-TTCCTTCCAACTCCAGAAAACAAG, R-CTAGATCGTCCAGAGGGCTTTC;

Mki67: F- GAGGAGAAACGCCAACCAAGAG, R-TTTGTCCTCGGTGGCGTTATCC;

Ccne1: F- AAGCCCTCTGACCATTGTGTCC, R- CTAAGCAGCCAACATCCAGGAC;

Ccna2: F-TTGTAGGCACGGCTGCTATGCT, R-GGTGCTCCATTCTCAGAACCTG;

Ccnb1: F-AGAGGTGGAACTTGCTGAGCCT, R- GCACATCCAGATGTTTCCATCGG;

Ccnb2: F-GCACTACCATCCTTCTCAGGTG, R- TGTGCTGCATGACTTCCAGGAC;

Col1a1: F- CCTCAGGGTATTGCTGGACAAC, R- CAGAAGGACCTTGTTTGCCAGG;

Col1a2: F- TTCTGTGGGTCCTGCTGGGAAA, R- TTGTCACCTCGGATGCCTTGAG;

Col2a1: F- GCTGGTGAAGAAGGCAAACGAG, R- CCATCTTGACCTGGGAATCCAC;

Col3a1: F- GACCAAAAGGTGATGCTGGACAG, R- CAAGACCTCGTGCTCCAGTTAG;

Irf7: F- CCTCTGCTTTCTAGTGATGCCG, R-CGTAAACACGGTCTTGCTCCTG;

Ifit1: F- TACAGGCTGGAGTGTGCTGAGA, R- CTCCACTTTCAGAGCCTTCGCA;

Isg15: F- CATCCTGGTGAGGAACGAAAGG, R- CTCAGCCAGAACTGGTCTTCGT;

Oasl2: F- CCAAAACGAGGTCGTCAGGAAC, R- AGCCACCTGTTCCCATCCCTTT;

Gapdh: F-CATCACTGCCACCCAGAAGACTG, R-ATGCCAGTGACTTCCCGTTCAG.

### 2.4 Statistics

RNA-seq and RT-qPCR graphs are shown as mean ± standard deviation (STD) with the indicated number of replicates (n = 3 to n = 6 biological replicates). Each RT-qPCR replicate was measured as a technical duplicate. All numerical analyses of the results were evaluated for statistical significance using standard algorithms in Prism (GraphPad Software, San Diego, CA) at probability values below 0.05 (p < 0.05). Significance was determined by Student's t-test.

## 3 Results

Modulations in the expression of cell cycle- and extracellular matrix (ECM)-related genes in proliferating versus quiescent MSCs under dynamic strain.

**Fig. 1:**
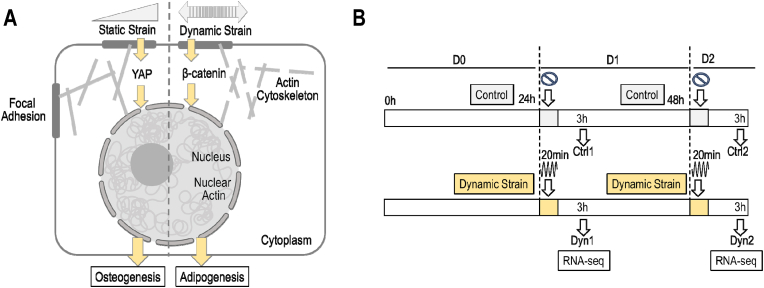
**Experimental plan. (A)** Schematic diagram showing mechanistic differences in mechanosensitivity depending on dynamic versus static strain. Entry of β-catenin into the nucleus due to dynamic strain is dependent on actin transport, while YAP nuclear entry depends on a static strain delivering force to open nuclear pores. **(B)** Overview of the experimental design. MSCs were exposed to dynamic strain (200 cycles × 2 % for 20 min) for one or two days in culture. Cells were harvested at 3 h after treatment for RNA isolation and RNA-seq analysis.

Dynamic strain increases cellular stiffness, re-structures the actin-cytoskeleton, promotes nuclear accumulation of G-actin and β-catenin, but does not stimulate nuclear YAP1 levels [27](Fig. 1A). This study examined how nuclear gene expression is modified upon induction of dynamic strain and actin-related nuclear access of β-catenin to mediate specific mechanoresponses in MSCs. Because MSCs may respond differently to mechanical stimuli in proliferative versus quiescent states, we examined how MSCs respond to dynamic strain during two-day time course when cells transition from a proliferative to a quiescent state. In both stages, we investigated the transcriptomic effects of applying dynamic strain (200 cycles × 2 % for 20 min) relative to untreated cultures at one or two days after plating of mouse bone marrow stem cells (Fig. 1B). Cells were harvested at 3 h after treatment for RNA isolation and RNA-seq analysis. When cells experience dynamic strain, they exhibit a strain-responsive cytoskeletal reorganization of β-actin (Suppl. Fig. 1). This reorganization occurs concomitant with shuttling of β-catenin into the nucleus based on images obtained by immunofluorescence microscopy and phalloidin staining [27]. Dynamic strain increases F-actin and cell stiffness (22 % increase in cell modulus as measured by AFM) at 3 h after strain application [27]. The RNA-seq analyses presented here reveal the transcriptome changes that occur in response to these modifications in cell morphology.

**Fig. 2:**
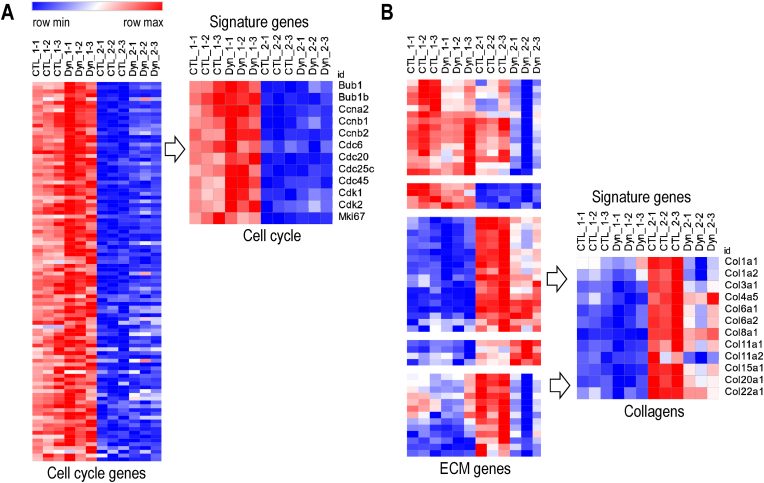
**Downregulation of proliferation markers in MSCs at day 2 in culture. (A)** Heatmap analysis using annotated cell cycle genes (n = 100; far left) shows that MSCs at day 1 express higher levels of RNA for cell cycle genes compared to day 2, regardless of dynamic strain. A breakout for selected cell cycle genes (n = 12; center right) is shown for comparison; these genes were selected based on well-established roles in cell cycle control (by GO terms) and similarities in expression patterns as observed by hierarchical clustering in preliminary heatmaps **(B)** Heatmaps for representative ECM-related genes (e.g., collagens, MMPs, TIMPs, non-collagenous proteins). (center right). A breakout for representative collagens (n = 12) shows that collagen mRNA expression increases on day 2 compared to day 1, but dynamic strain reduces collagen expression.

**Fig. 3:**
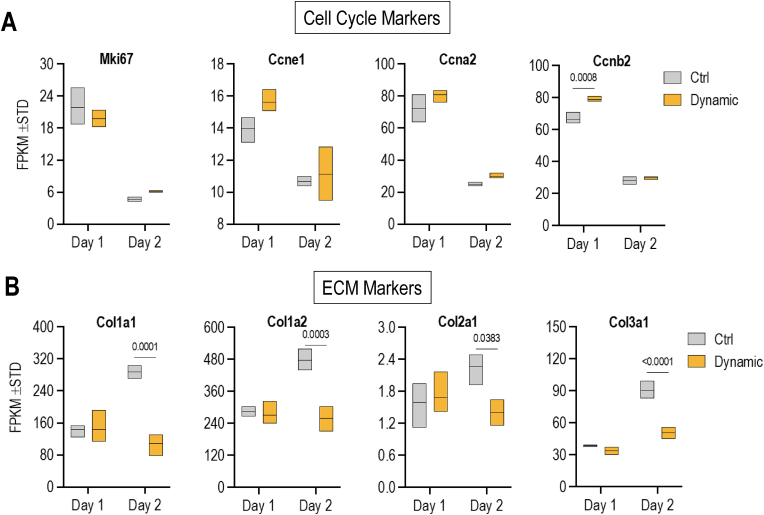
**Dynamic strain does not affect MSC proliferation (A)** Box & whisker plots show mRNA levels for the indicated proliferation markers are expressed as FPKMs. Levels were reduced in the second day of the experiment when MSCs become contact inhibited and reach quiescence. There were no significant differences between control and dynamic strain groups, indicating that dynamic strain has no effect on cell proliferation markers. **(B)** Extracellular matrix (ECM) markers (expression in FPKM) show a modest reduction on the second day after dynamic strain compared to control group (RNA-seq data).

RNA-seq data were first subjected to supervised analysis of known markers for cell proliferation and extracellular matrix deposition (Fig. 2, Fig. 3). Heatmap analysis for representative proliferation-related genes (n = 100; gene ontology category: cell cycle) shows that all proliferation specific genes we selected show downregulation from day 1 to day 2 in control cells (Fig. 2). Conversely, heatmap analysis for representative ECM related genes (a supervised set of n = 100 collagens and non-collagenous proteins that was derived from a larger set of genes selected by gene ontology analysis) shows upregulation in control cells on day 2 (Fig. 2). This finding indicates that bone marrow-derived MSCs transition from a proliferative stage into a quiescent stage, consistent with previous findings in adipose-tissue derived MSCs [49]. Hence, our cell culture model is sufficiently robust to address the question whether the proliferative versus non-proliferative state of MSCs can affect mechanoresponsiveness of genes.

Analysis of individual genes detected by RNA-seq shows reduced mRNA levels of the proliferation marker MKI67 and the cell cycle stage specific markers cyclin E1 (CCNE1; G1/S phase transition), cyclin A2 (CCNA2; S/G2 phase) and cyclin B1 or B2 (CCNB1 and CCNB2; G2/M) (Fig. 3A). The reduction of these markers (Fig. 3A) and other cell cycle markers selected based on gene ontology and/or similarities in gene expression (Fig. 2A) is independent of dynamic strain. Thus, MSC proliferation is not modulated by dynamic strain under our experimental conditions. Analysis of individual ECM proteins shows upregulated expression of multiple collagen mRNAs (e.g., Col1a1, Col1a2, Col3a1) under control conditions in the absence of strain (Fig. 3B). Application of dynamic strain significantly attenuates the upregulation of these collagen mRNAs (Fig. 3B), as well as other collagen mRNAs (Fig. 2B) and non-collagenous extracellular matrix related genes (Suppl. Table S1 & S2).

### 3.1 Dynamic strain modulates expression of distinct sets of genes at different days in culture

**Fig. 4:**
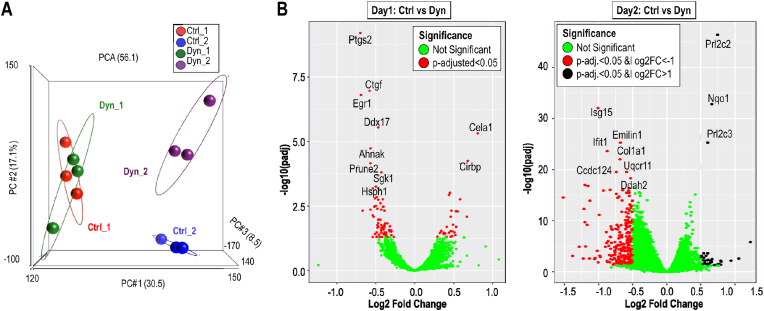
**Global changes in MSC transcriptomes after dynamic strain (A)** Principal component analysis (PCA) results show differences in MSC transcriptomes under control and dynamic strain conditions on day1 (red vs green) and day 2 (blue vs purple). **(B)** Volcano plots show differentially genes at day 1 (center left) or day 2 (right) after dynamic strain based on DEseq2 analysis. In proliferating cells at day1, there are 105 differentially expressed genes upon application of dynamic strain (adj. p < 0.05). However, none of these genes exhibit expression changes of more than two-fold (i.e., |log2FC|>1. There are >200 differentially expressed genes that are differentially expressed at day 2 that are both statistically significant (adj. P < 0.05) and have a meaningful biological change (|log2FC|>1). (For interpretation of the references to color in this figure legend, the reader is referred to the Web version of this article.)

To understand how MSCs respond to dynamic strain on proliferative (day 1) versus quiescent stages (day 2), we performed gene expression analyses using RNA-seq (Fig. 4) of cells that were treated for one or two days with 20 min of dynamic strain (see Fig. 1). We performed principal component analysis (PCA) with a matrix of all genes that were detected on either day 1 or 2, with or without strain treatment. On each day we detected a similar number of genes (day 1: n = 12,574 & day 2: n = 12,712 genes; n = 43,346 total annotated mouse genes). The PCA plots show that the transcriptomes of proliferating MSCs (day 1) are very different from quiescent MSCs (Fig. 4A). Data dots for day 1 versus day 2 are far apart in multidimensional data space and PC1 appears to correlate with differences in cell proliferation (Fig. 4A). Furthermore, control MSCs and MSCs subjected to dynamic strain have very similar transcriptomes at day 1 (i.e., data dots cluster closely together), but not on day 2 (Fig. 4A). Hence, the gene expression profiles of proliferating and quiescent cells are very different, and dynamic strain has more pronounced global transcriptomic effects in quiescent cells (day 2) compared to proliferating cells (day 1).

**Fig. 5:**
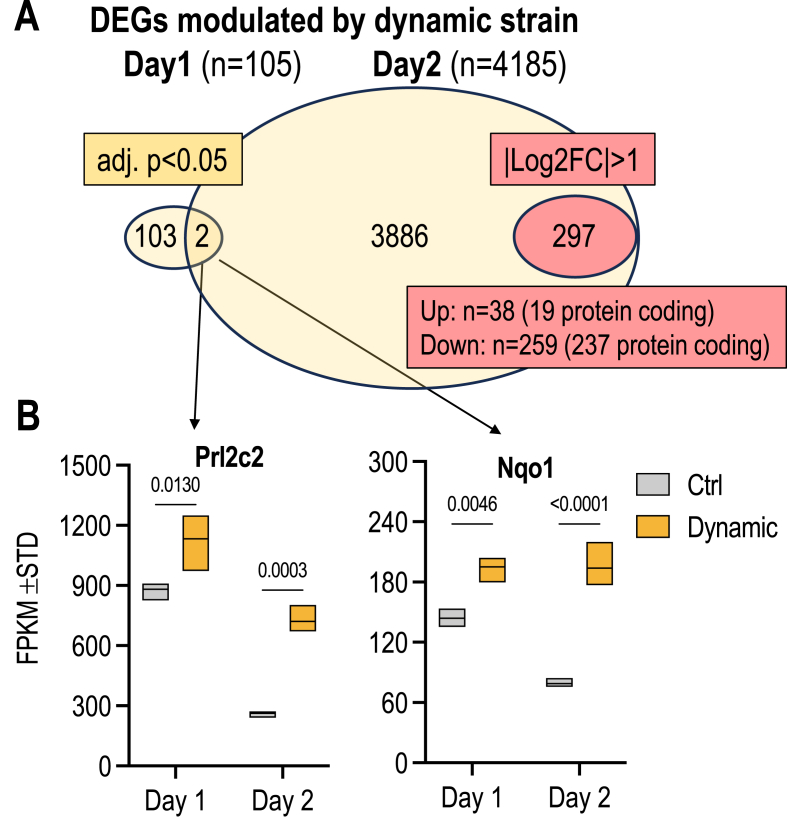
**Dynamic strain affects different sets of genes in MSCs depending on the culture stage. (A)** The Venn diagram that compares differentially expressed genes (DEGs) on both day1 and day 2, reveals that only two genes (Prl2c2 and Nqo1) are regulated by dynamic strain on both days. Yellow ovals show differentially expressed genes based only on p-value (adj. p < 0.05), while pink ovals show genes with |log2FC|>1 that are only detected on day2. Differentially expressed genes on day2 include 19 upregulated and 237 downregulated protein coding genes as indicated. **(B)** Box & whiskers plot of RNA-seq data (in FPKM) for Prl2c2 and Nqo1 (n = 3 per sample) with associated p-values. The Venn diagram in Panel B was used as the basis for STRING analysis presented in Supplementary Figures S4 to S7. (For interpretation of the references to color in this figure legend, the reader is referred to the Web version of this article.)

Differentially expressed genes (DEGs) were identified by DEseq2 analysis and visualized by volcano plots and Venn diagrams (Fig. 4, Fig. 5, Suppl. Fig. S2 & Suppl. Tables S1 & S2). Filtering for genes with adjusted p-values (p < 0.05) established that there are few DEGs in proliferating MSCs (day 1; n = 105) and many more DEGs in quiescent MSCs (day 2; n = 4185) after application of dynamic strain in MSCs (Fig. 4, Fig. 5A). The volcano plots show the top 10 up or downregulated genes (based on |Log2(FC)| (Fig. 4B). Further filtering for numerical changes (|log2|>1) established that all of the DEGs at day 1 show strain-dependent changes in gene expression are less than 2-fold (Fig. 4, Fig. 5A). In contrast, there are 297 DEGs at day 2 that change by 2 fold or more (Fig. 5A), including 38 upregulated DEGs and 259 downregulated DEGs. The limited number of DEGs on day 1 and greater number of DEGs on day 2 is consistent with the PCA analysis (see Fig. 4A).

Remarkably, only 2 genes are in common on both days and these gene exhibit statistically significant but numerically modest upregulation (i.e., Prl2c2 and Nqo1) (Fig. 5A & B, Suppl. Figs. S2-S4). Prl2c2 is a member of a larger group of prolactin related genes, five of which are expressed at detectable levels in MSCs (i.e., Prl2c2, Prl2c3, Prl2c5; FPKM>1), while Nqo1 is co-expressed with the related gene Nqo2 (Suppl. Fig. S5). Interestingly, there is only a single human PRL gene and its encoded protein decreases osteoblast proliferation [56]. Comparison of expression patterns among Prl2c members shows that Prlc1 and Prlc5 are expressed at much lower levels, while Prl2c2 and Prl2c3 exhibit relatively high expression; similar to Prlc2, Prl2c3 is expressed on day 2 and exhibits a numerical trend (p = 0.10) at day 1 (Suppl. Figs. S4 & S5). Compared to Nqo2, Nqo1 is expressed at 10-fold higher levels and strain responsive, whereas Nqo2 is not (Suppl. Figs. S5). Thus, dynamic strain selectively modulates the levels of two proteins (Prlc2 and Nqo2) that have not yet been extensively explored in bone.

To understand what broader gene expression programs were activated on day 2 (n = 297 DEGs), we performed STRING protein network analysis for protein coding genes (n = 256) that are either upregulated (n = 19) or downregulated (n = 237) by dynamic strain on day 2 (Suppl. Figs. S6 & S7, respectively). On day 2, strain enhances expression of a small set of genes (n = 19) including several genes associated with oxidative stress that include Nqo1, Prl2c2, Prl2c3) (Suppl. Fig. 6). Dynamic strain diminishes the expression of 237 genes that are associated with interferon (IFN) responsiveness (e.g., Irf7, Oasl2, Isg15), ECM (e.g., Col1a1, Mmp13, Col15a1), translation (e.g., Rplp1, Rplp2), transcription factors (e.g., TFs Junb, Jund, Cebp), and ephrin signaling (e.g., Ephb2, Efna3) (Suppl. Fig. S7). Collectively, these data show that dynamic strain results in selective changes in the transcriptome of MSCs and strain induced changes in their phenotypic properties related to IFN and Ephrin signaling, as well as transcription and translation.

**Fig. 6:**
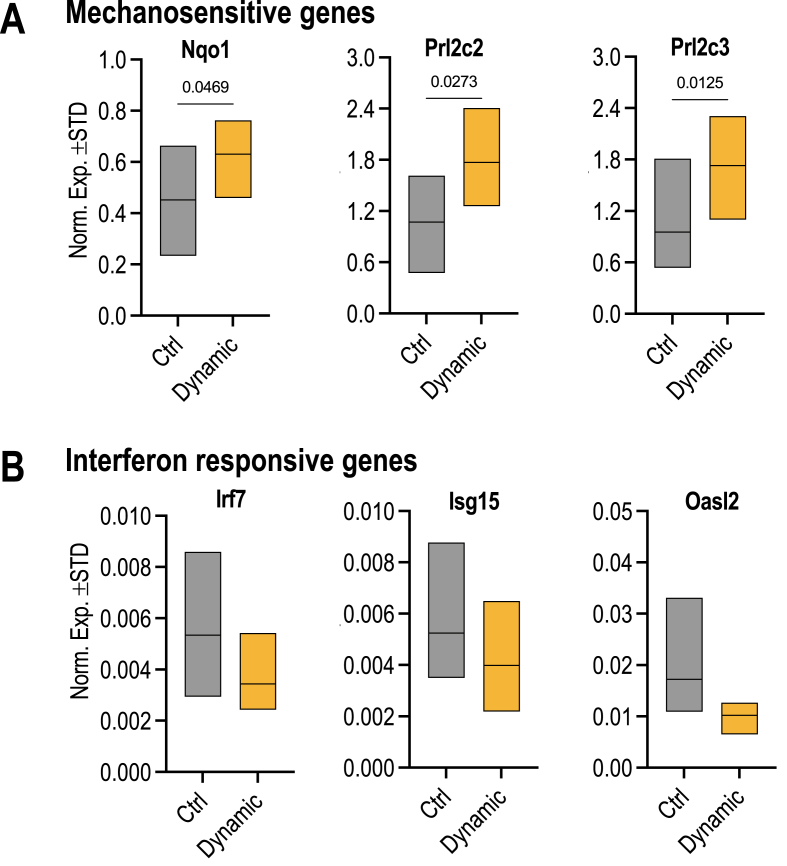
**RT-qPCR analysis of genes associated with oxidative responses and interferon signaling. (A)** RT-qPCR analysis of RNA isolated from cells subjected to control conditions (gray) or dynamic strain (yellow) in MSCs. The data show oxidative stress related genes (Nqo1, Prl2c2, and Prl2c3) that are significantly increased due to dynamic strain. **(B)** RT-qPCR analysis of the same samples shows that interferon related genes (Irf7, Isg15, and Oasl2) each show a numerical trend towards reduced expression upon mechanical dynamic strain**.** Graphs show data from biological triplicates that were each analyzed as technical duplicates that counted as one observation (n = 3, mean ± STD). Student's t-test was used to determine statistical significance (shown above the bar graphs in Panel A). Numerical trends observed in Panel B did not yet reach significance (p > 0.05), even though the corresponding expression data obtained by RNA-seq are significant. (For interpretation of the references to color in this figure legend, the reader is referred to the Web version of this article.)

We validated the modulations in expression of oxidative stress related genes (i.e., Nqo2, Prl2c2 and Prl2c3), as well as select interferon responsive genes (i.e., Irf7, Oasl2 and Isg15) after dynamic strain by RT-qPCR analysis using triplicate samples from two independent biological experiments (n = 6 total) (Fig. 6) to replicate the earlier findings obtained by RNA-seq analysis. The RT-qPCR results establish that Nqo2, Prl2c2 and Prl2c3 each exhibit statistically significant changes in gene expression (p < 0.05 in each case), while the Irf7, Oasl2 and Isg15 genes each exhibit numerical trends (i.e., p-values of, respectively, 0.11, 0.22 and 0.07), consistent with the activation of a larger program of interferon responsive genes in which Irf7, Oasl2 and Isg15 are often coregulated (see Suppl. Fig. S7).

**Fig. 7:**
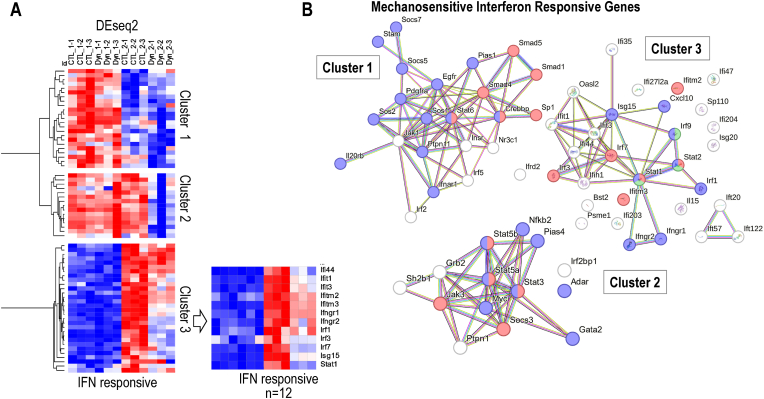
**Heatmap analysis of RNA-seq data for genes associated with interferon signaling. (A)** A supervised heatmap was generated using the same RNA-seq samples as discussed in Fig. 5 except that only genes involved in interferon signaling were used in the analysis. The heatmap shows three main clusters that differ in patterns of expression: Cluster 1 encompasses IFN related genes expressed primarily expressed in proliferating cells that are not modulated by dynamic strain; Cluster 2 shows genes constitutively expressed on both day 1 and day 2, but that are only modulated by dynamic strain on day 2; Cluster 3 includes genes expressed primarily on day 2 and that are modulated by dynamic strain. **(B)** STRING diagrams of genes contained within Clusters 1, 2 and 3 as indicated. The nodes are colored based on gene ontology terms. Cluster 1: proteins involved in JAK/STAT signaling (purple) and DNA binding (red); Cluster 2: proteins involved in STAT/PI3K signaling (red) and DNA binding (purple); Cluster 3: Type I interferon signaling (red), Type II interferon signaling (purple) and interferon responsive ISGF3 transcriptional complex (green). (For interpretation of the references to color in this figure legend, the reader is referred to the Web version of this article.)

To understand the molecular interplay between dynamic strain and interferon responsive genes in greater detail, we performed hierarchical clustering based on gene expression for representative genes linked to interferon signaling based on gene ontology terms. Heatmap analysis shows that there are three types of interferon signaling related genes that show differential expression with respect to temporal stage or dynamic strain in our data set. One set of genes (Cluster 1) is only expressed at day 1 and is not modulated by dynamic strain. Cluster 2 genes are expressed on both day 1 and day 2 but are only downregulated by dynamic strain on day 2. Cluster 3 genes are only expressed at day 2 and their mRNA levels are collectively decreased on day 2 (Fig. 7). Hence, dynamic strain selectively decreases expression of interferon related genes when cells are reaching a confluent, quiescent state.

**Fig. 8:**
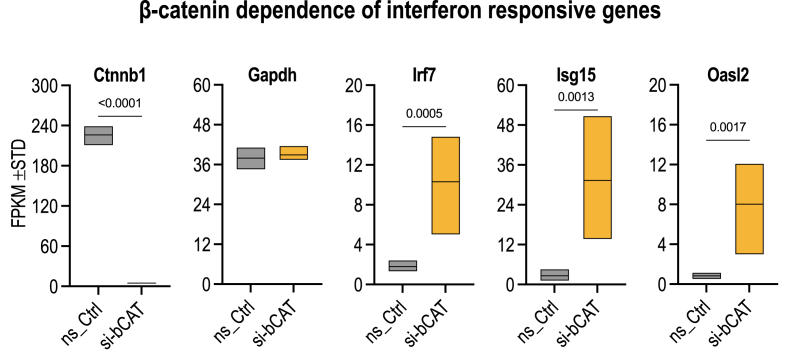
**Depletion of β-catenin reduces expression of genes involved in interferon signaling.** The graphs show data on gene expression (in FPKM) of MSCs treated with non-silencing RNA (ns-RNA, gray boxes) or silencing RNA for β-catenin (yellow boxes). The box & whisker plots show that β-catenin/Ctnnb1 mRNA is quantitatively depleted by the siRNA, while the mRNA levels for the housekeeping gene GAPDH do not change biologically relevant changes.The mRNA levels of three representative IFN related genes (i.e., Irf7, Oasl2 and Isg15; in FPKMs) exhibit statistically significant increased expression upon loss of β-catenin (p-values based on n = 6 biological replicates). (For interpretation of the references to color in this figure legend, the reader is referred to the Web version of this article.)

Because dynamic strain transports β-catenin to the nucleus, the possibility arises that the decrease in expression of IFN genes is due to changes in the nuclear levels of β-catenin. We tested whether β-catenin is rate-limiting for IFN gene expression by analyzing the effects of depleting β-catenin mRNA levels in MSCs using siRNA (Fig. 8). MSCs treated with siRNA for β-catenin/Ctnnb1 exhibit a ∼50 fold reduction in Ctnnb1 mRNA levels, indicating that the siRNA is very effective. The results show that the expression of three representative IFN-related genes (i.e., Irf7, Oasl2 and Isg15) is dramatically increased upon loss of β-catenin, reflected by 5- to 10-fold enhancement in mRNA levels for these genes. These data indicate that the WNT/β-catenin pathway controls expression of genes related to interferon signaling in MSCs.

## 4 Discussion

In this study, we established differences in the molecular response of MSCs to dynamic strain depending on whether the cells are proliferating or quiescent. Actively proliferating MSCs typically do not have a dense collagenous matrix and limited cell-cell contact. We show that dynamic strain modulates expression of >4000 genes in quiescent MSCs (day 2; n = 4185) and expression for ∼7 % of these genes (n = 297) is increased by 2-fold or more. In contrast, expression of only ∼100 genes (day 1; n = 105) is modulated in proliferating cells, and the fold change relative to controls is modest (FC < 2). Dynamic strain in quiescent MSCs enhances the expression of genes involved in oxidative stress (e.g., Nqo1, Prl2c2, Prl2c3) and inhibits transcript levels of interferon responsive genes (e.g., Irf7, Oasl2 and Isg15). These findings indicate that dynamic strain provokes distinct biological programs in uncommitted MSCs. The differences in MSC responsiveness to dynamic strain on day 1 versus day 2 suggests that dynamic strain is more effective in regulating gene expression when cells stop proliferation and are connected through cell-cell contact and/or interactions with the collagenous ECM.

Dynamic strain controls expression of several genes (e.g., Nqo1, Prl2c2 and Prlc3) that are associated with anti-oxidative responses and signaling pathways related to somatostatin and prolactin ligands. The stimulation of these oxidative stress related genes suggests that dynamic strain may elevate reactive oxygen species (ROS), which are linked to mitochondrial function and physical activity [57]. Human prolactin is encoded by a single gene PRL and has been shown to decrease osteoblast proliferation [56]. The mouse genome has multiple PRL genes (n = 23), and our data show that Prl2c2 and Prl2c3 are the only members of this class that are expressed in mouse bone marrow derived MSCs. The functional significance of the divergence in prolactin gene copies in human and rodent species has not been fully explored. Prolactin is a member of a larger class of peptide hormones that act as cytokines in some biological contexts, and these peptides interact with and signal through the prolactin receptor (Prlr). Our RNA-seq data indicate that MSCs do not express the Prlr receptor, suggesting that the increase in Prl2c2 and Prlc3 expression may serve a proliferation-suppressive paracrine function to an as yet unknown cell type that expresses this Prlr receptor. While prolactin-related proteins have a clear endocrine function through abundant expression in the pituitary gland, systemic factors could have short half-lives. Expression of Prl2cl and Prl2c3 in MSCs would then provide a local source of PRL proteins in the bone marrow environment. We also observed upregulation of Nqo1 gene which is an FAD-binding protein with NAD(P)H dehydrogenase activity that acts as a cytoplasmic 2-electron reductase to reduce quinones to hydroquinones. Modulations in the expression of Prl and Nqo1-related genes by dynamic strain may reflect an anti-oxidative response linked to cell metabolism, autophagy and/or apoptosis [58].

The most pronounced change in gene expression in quiescent MSCs (day 2) upon dynamic strain is the suppression of multiple interferon (IFN) responsive genes (e.g., Irf7, Oasl2 and Isg15). We find that IFN responsive genes are activated in MSCs depleted of β-catenin using siRNAs. Therefore, it is plausible that dynamic strain may induce nuclear β-catenin to suppress IFN responsive genes in MSCs. Because dynamic strain favors adipogenesis over osteogenesis [27], the expression of these IFN genes may block adipogenesis and therefore favor osteogenesis as part of a molecular mechanism that regulates early stages of lineage-commitment in post-proliferative MSCs. This interpretation is consistent with previous findings showing that interferon γ (IFNG) signaling blocks adipogenesis [59]. There is a surprising paucity of studies on the upstream effects of Wnt/β-catenin on interferon γ signaling in mesenchymal cells. One study examined the downstream effects of interferon γ on Wnt/β-catenin signaling [60] in non-bone cells, and a second set of studies showed that interferon-β (IFNB1) impairs early stages of mineralization in MSCs [21,61,62]. The novelty of the current work is reflected by our finding that both dynamic strain and Wnt/β-catenin signaling suppress the interferon response, providing new experimental evidence for potential crosstalk between these two principal signaling pathways.

The JAK/STAT-dependent interferon response correlates with induction of osteoblastogenesis, and this pathway is suppressed by the epigenetic master suppressor EZH2 that blocks osteogenesis [1,42,63]. The suppression of this pathway by dynamic strain is consistent with the inhibition of osteogenesis by dynamic strain and our previous observation that dynamic strain increases nuclear accumulation of β-catenin to support adipogenesis [27]. We note that nuclear β-catenin also enhances Ezh2 levels [28], which favors the uncommitted self-renewing state of MSCs. Upregulation of β-catenin in quiescent cells in response to dynamic strain may increase Ezh2 activity, suppress expression of ECM genes and potentially IFN responsive genes.

The role of nuclear β-catenin in maintaining the uncommitted self-renewing state of MSCs may be distinct from its role during osteogenesis. In proliferating MSCs, β-catenin may not only activate Ezh2 but also Myc and Cyclin D1 (Ccnd1) to support cell cycle progression, whereas this pathway is blocked by Cdk inhibitors in quiescent cells. This mechanism is similar to what is seen with FGF2/FGFR signaling. In stem cells, FGF2 is a mitogen, but in non-proliferative cells it is a morphogen that induces differentiation because CDK inhibitors are expressed at high levels in post-proliferative cells. The latter blocks the proliferative effects of both WNT/β-catenin and FGF2/FGFR1/MAPK, permitting repurposing of both signaling pathways towards differentiation [64,65].

Limitations of the present study are that we examined dynamic strain at early stages of cell culture when MSCs switch from a proliferative state to a confluent state. We only tested a single regimen of repetitive strain cycles on two consecutive days. It will be of interest to assess what happens upon varying the protocol for applying dynamic strain and/or simultaneously inducing either the osteogenic or adipogenic phenotype. While our study shows that both dynamic strain and β-catenin suppress the interferon response, it remains to be established what happens when β-catenin is depleted simultaneously with dynamic strain treatment. Furthermore, our analyses are restricted to mRNA analyses and will require validation of changes in the encoded proteins by western blots and immunofluorescence microscopy in future studies.

In conclusion, the novelty of this study is the demonstration that the biological and molecular effects of dynamic strain on MSCs depend on the level of proliferation versus a non-proliferating quiescent state. In proliferating cells, we do not observe a major change in the overall transcriptome upon dynamic strain. However, in quiescent MSCs we observe major phenotypic changes in the cells upon dynamic strain as reflect by changes in the transcriptome. The most prominent change in gene expression is the suppression of interferon γ responsive genes and this suppression may be mediated by β-catenin. These new findings suggest a novel type of dynamic strain-related crosstalk between Wnt/β-catenin signaling and the interferon γ pathway. Importantly, interferon γ signaling suppresses adipogenesis. Therefore, our data are consistent with a regulatory model in which dynamic strain may activate β-catenin to suppress interferon responsive genes and stimulate adipogenic differentiation.

## Author contributions

PD prepared the first draft of this manuscript in consultation with GSS, BCJvdE, JPTMvL, and AJvW. PD performed experimentation and EAL assisted with the bioinformatic analysis. All authors discussed the draft, provided conceptual input and edited the paper. PD & AJvW finalized the draft for submission. AJvW obtained the main source of funding for this study and BCJvdE provided auxiliary funding.

## Funding

These studies were supported by 10.13039/100000069National Institute of Arthritis and Musculoskeletal and Skin Diseases (10.13039/100000069NIAMS) grant R01-AR049069 (to AJvW), as well as internal funding of Erasmus University Medical Center (to BCJvdE).

## Declaration of competing interest

The authors have no conflicts of interest to declare. All co-authors have seen and agree with the contents of the manuscript and there is no financial interest to report.

## Data Availability

Relevant RNA-seq data are provided as supplementary tables

## Appendix A Supplementary data

**Supplementary Figure S1**: Formalin (4 %)-fixed cultured on silicone membranes were stained for Alexa Fluor 488-conjugated phalloidin and DAPI to reveal the cytoskeletal and nuclear morphology of bone marrow MSCs subjected to dynamic strain. The micrographs show stiffer cytoskeletal elements upon dynamic strain, similar to previously reported findings.

**Supplementary Figure S2**: Tabular representation of differentially expressed (DE) genes in response to dynamic strain at day 1 or day 2 as identified by DESeq2. DE genes conformed to two distinct criteria (i.e., adjusted p < 0.05 and |log2FC|> 1 or 2, as indicated in above the columns. The two data rows show DE genes at day 1 (Dyn_1 vsCTL_1) or day 2 (Dyn_2 vsCTL_2).

**Supplementary Figure S3**: Box & whisker plots of representative differentially expressed genes at day 1 (in FPKM, p values based on n = 3 biological replicates.

**Supplementary Figure S4**: Box & whisker plots of representative differentially expressed genes at day 2 (in FPKM, p values based on n = 3 biological replicates.

**Supplementary Figure S5**: Box & whisker plots of genes comparing expression of various members of the Nqo or Prl2c family of genes (in FPKM, p values based on n = 3 biological replicates.

**Supplementary Figure S6**: STRING diagrams of genes that are upregulated by dynamic strain on day 2. The network shows connections between Nqo1 and Prl2c2 and several other proteins (pink circles), while the other genes are not part of the same network (open circles).

**Supplementary Figure S7**: STRING diagrams of genes that are downregulated by dynamic strain on day 2. These genes include a large group of interconnected IFN responsive genes, as well as proteins related to translation, transcription factors (TFs) ECM and ephrin signaling as indicated.

Multimedia component 1 Multimedia component 1

Multimedia component 2 Table of expression values for differentially expressed (DE) genes in response to dynamic strain at day 1 as identified by DESeq2 (Sheet #1). DE genes conformed to the criterion of statistical significance (i.e., adjusted p < 0.05) are presented in Sheet#2. No genes conformed to the criterion |log2FC|> 1 or 2. Multimedia component 2

Multimedia component 3 Table of expression values for differentially expressed (DE) genes in response to dynamic strain at day 2 as identified by DESeq2 (Sheet #1). DE genes conformed to two distinct criteria (i.e., adjusted p < 0.05 and |log2FC|>1 (see Sheet#2) or adjusted p < 0.05 and |log2FC|>2 (see Sheet #3). Multimedia component 3
