# Supplementary material for: Dynamic strain and β-catenin mediated suppression of interferon responsive genes in quiescent mesenchymal stromal/stem cells
Source: Biochem Biophys Rep. 2024 Oct 23;40:101847. doi: 10.1016/j.bbrep.2024.101847 (PMC11541450; doi:10.1016/j.bbrep.2024.101847)
Supplement: Multimedia component 1 [file mmc1.pptx]

## Slide 1
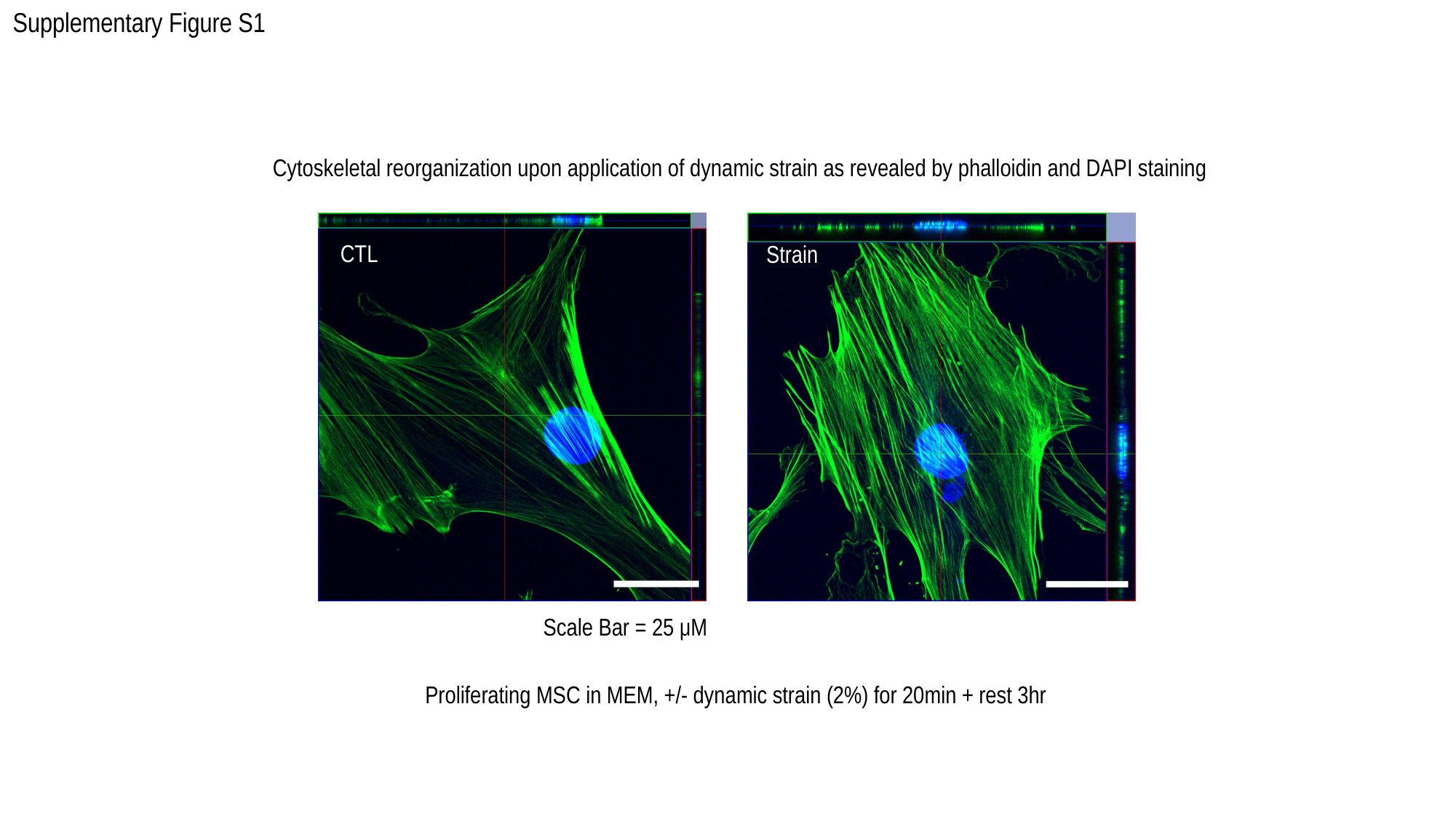

Supplementary Figure S1
Cytoskeletal reorganization upon application of dynamic strain as revealed by phalloidin and DAPI staining
Strain
CTL
Scale Bar = 25 μM
Proliferating MSC in MEM, +/- dynamic strain (2%) for 20min + rest 3hr

## Slide 2
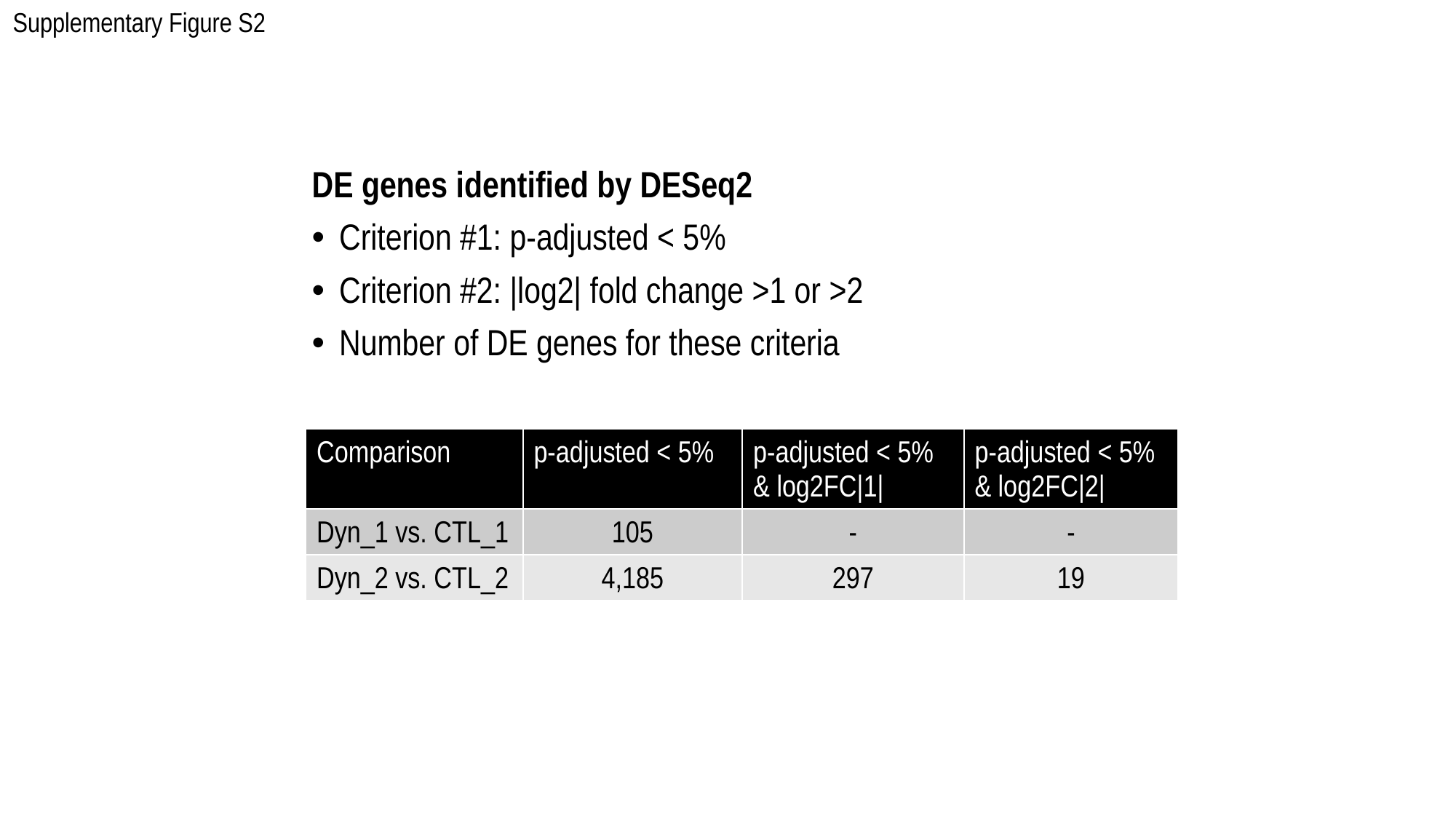

Supplementary Figure S2
DE genes identified by DESeq2
Criterion #1: p-adjusted < 5%
Criterion #2: |log2| fold change >1 or >2
Number of DE genes for these criteria
| Comparison | p-adjusted < 5% | p-adjusted < 5% & log2FC|1| | p-adjusted < 5% & log2FC|2| |
| --- | --- | --- | --- |
| Dyn\_1 vs. CTL\_1 | 105 | - | - |
| Dyn\_2 vs. CTL\_2 | 4,185 | 297 | 19 |

## Slide 3
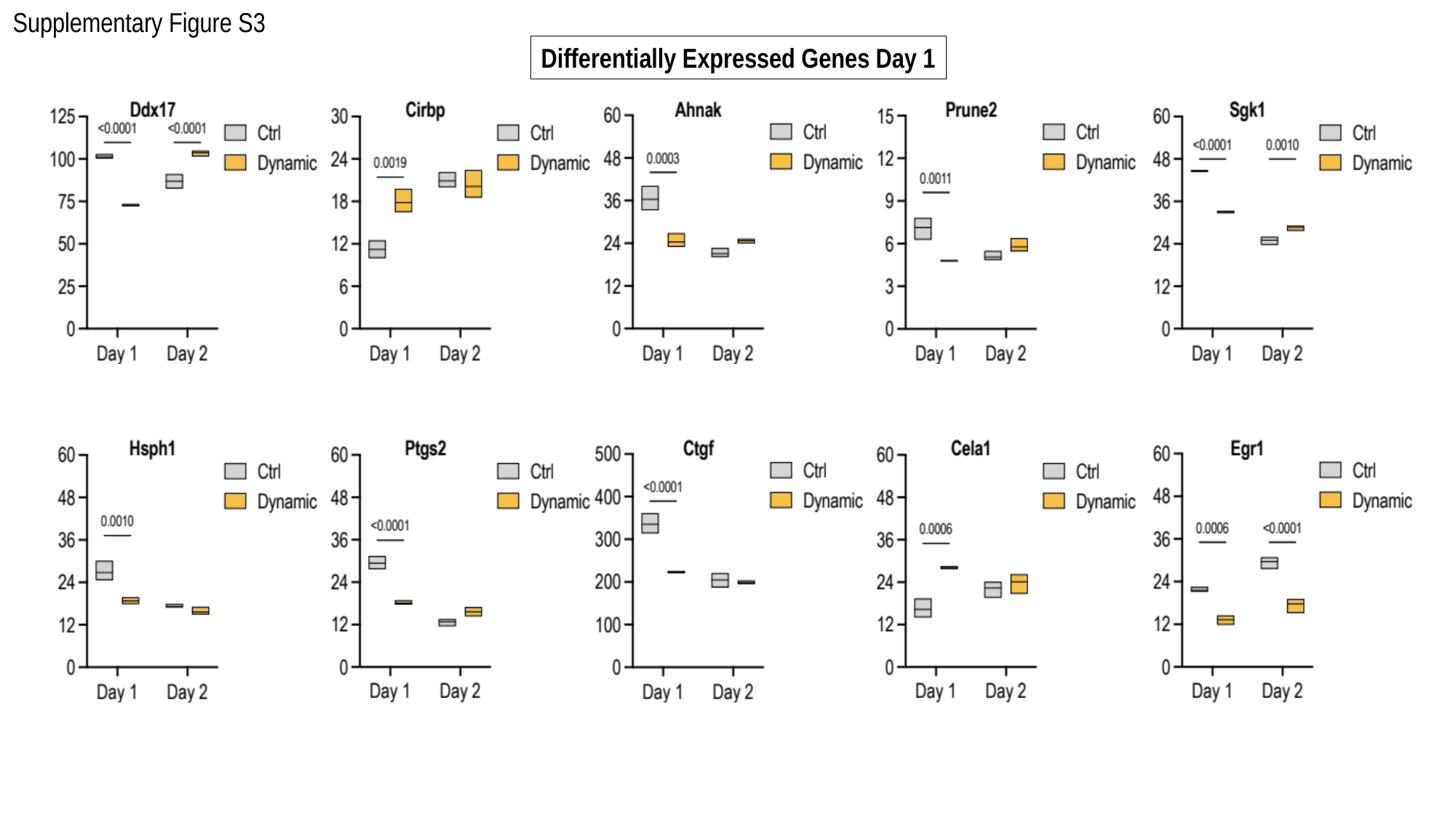

Supplementary Figure S3
Differentially Expressed Genes Day 1

## Slide 4
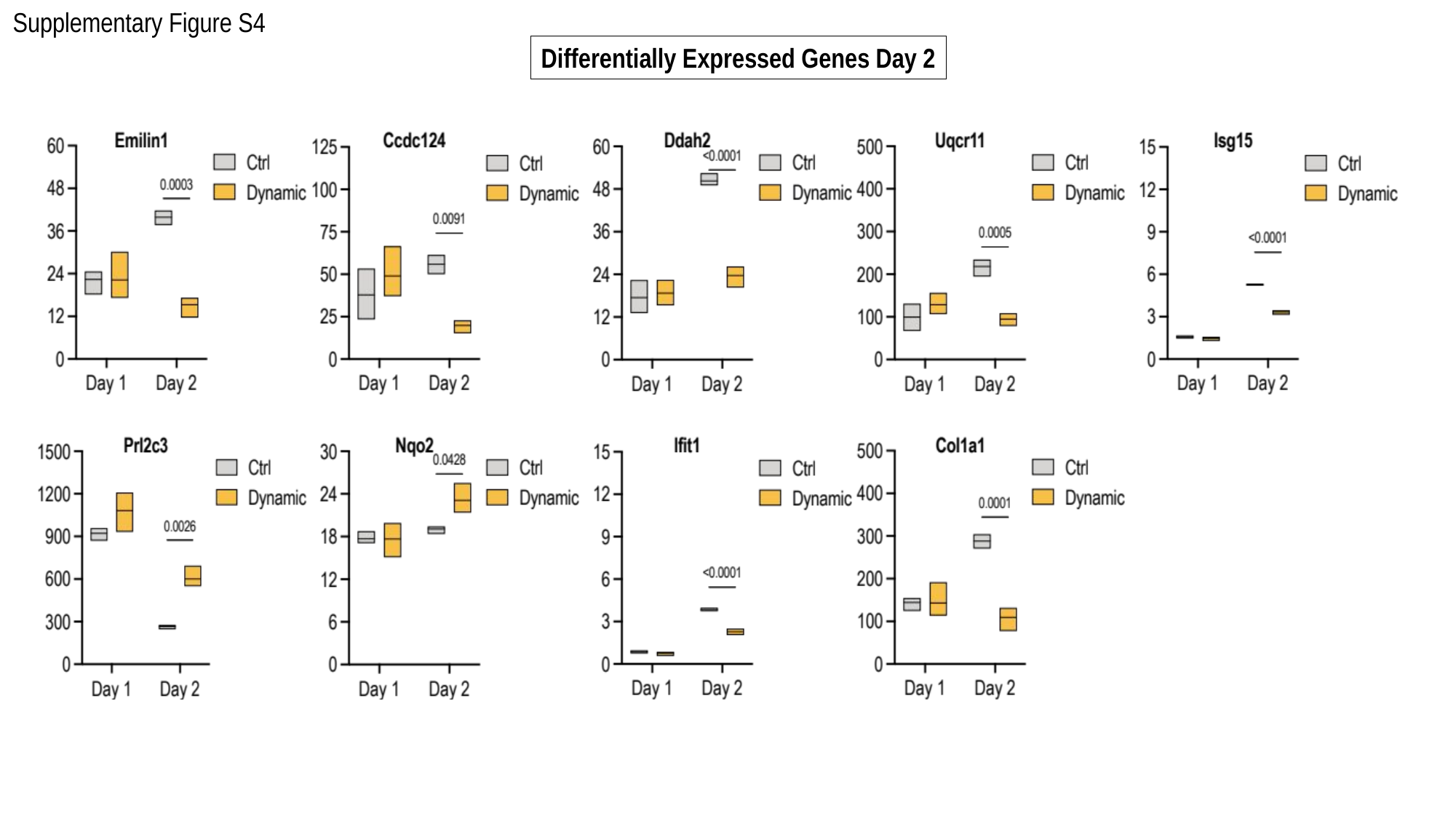

Supplementary Figure S4
Differentially Expressed Genes Day 2

## Slide 5
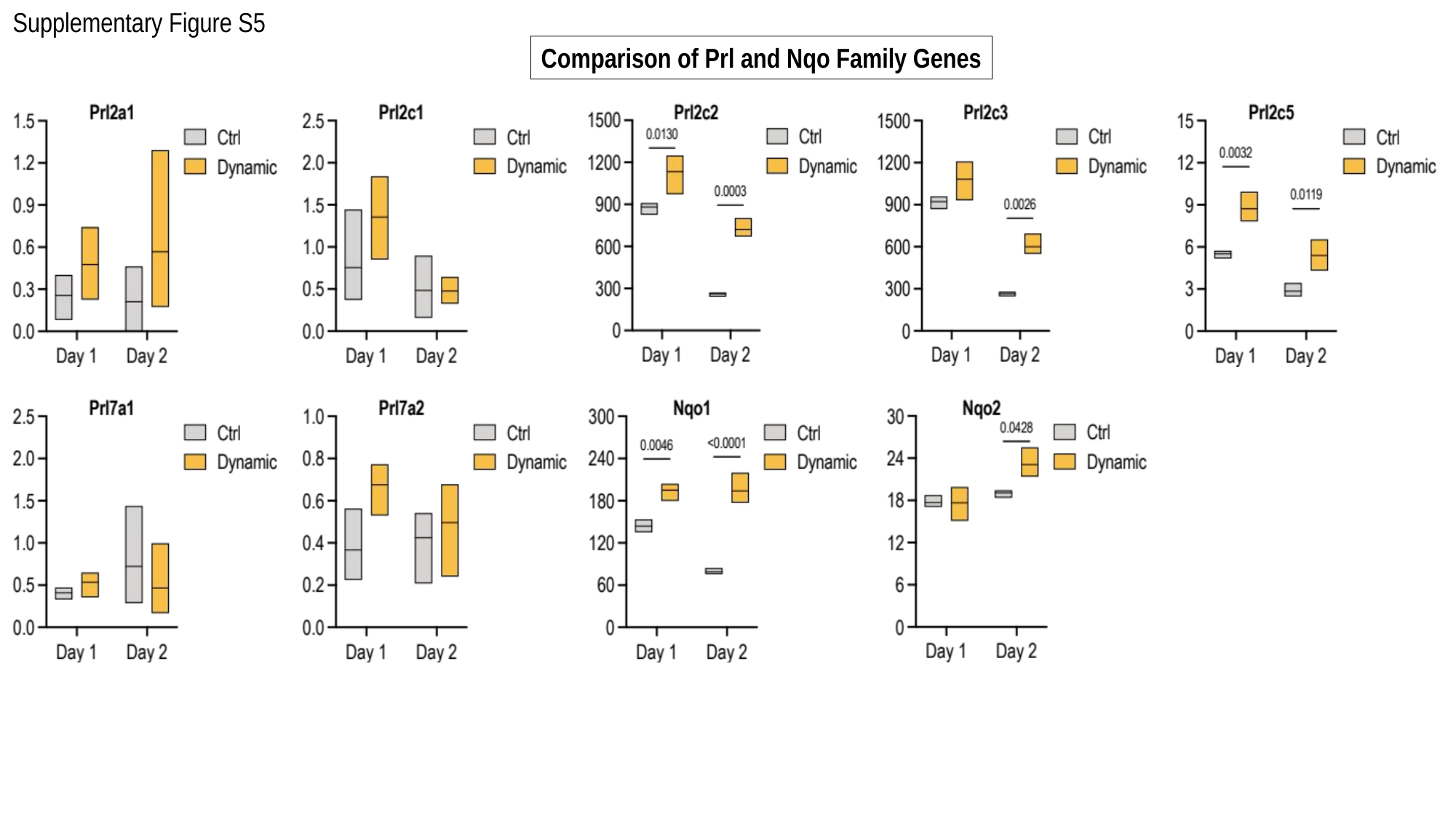

Supplementary Figure S5
Comparison of Prl and Nqo Family Genes

## Slide 6
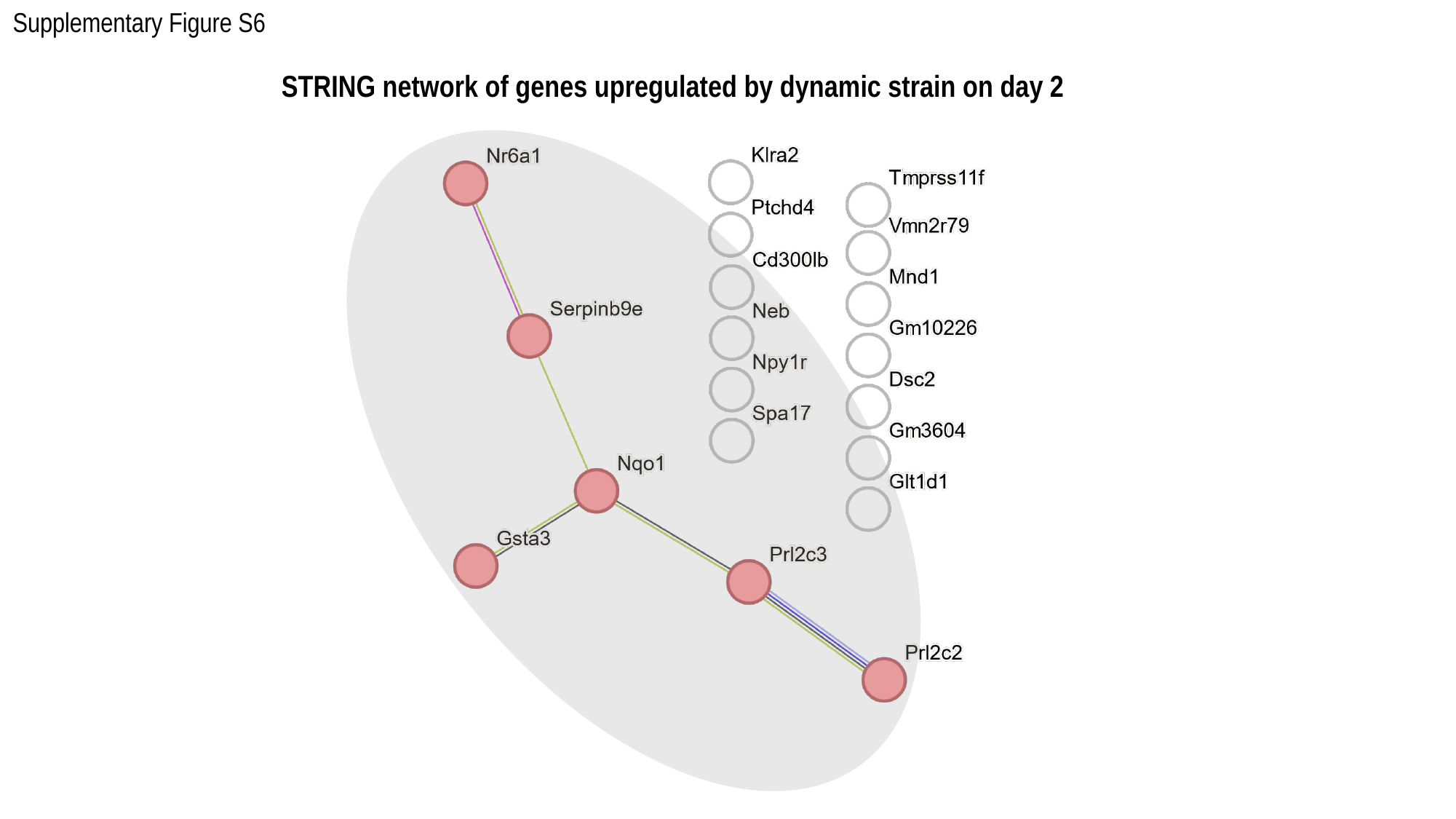

Supplementary Figure S6
STRING network of genes upregulated by dynamic strain on day 2

## Slide 7
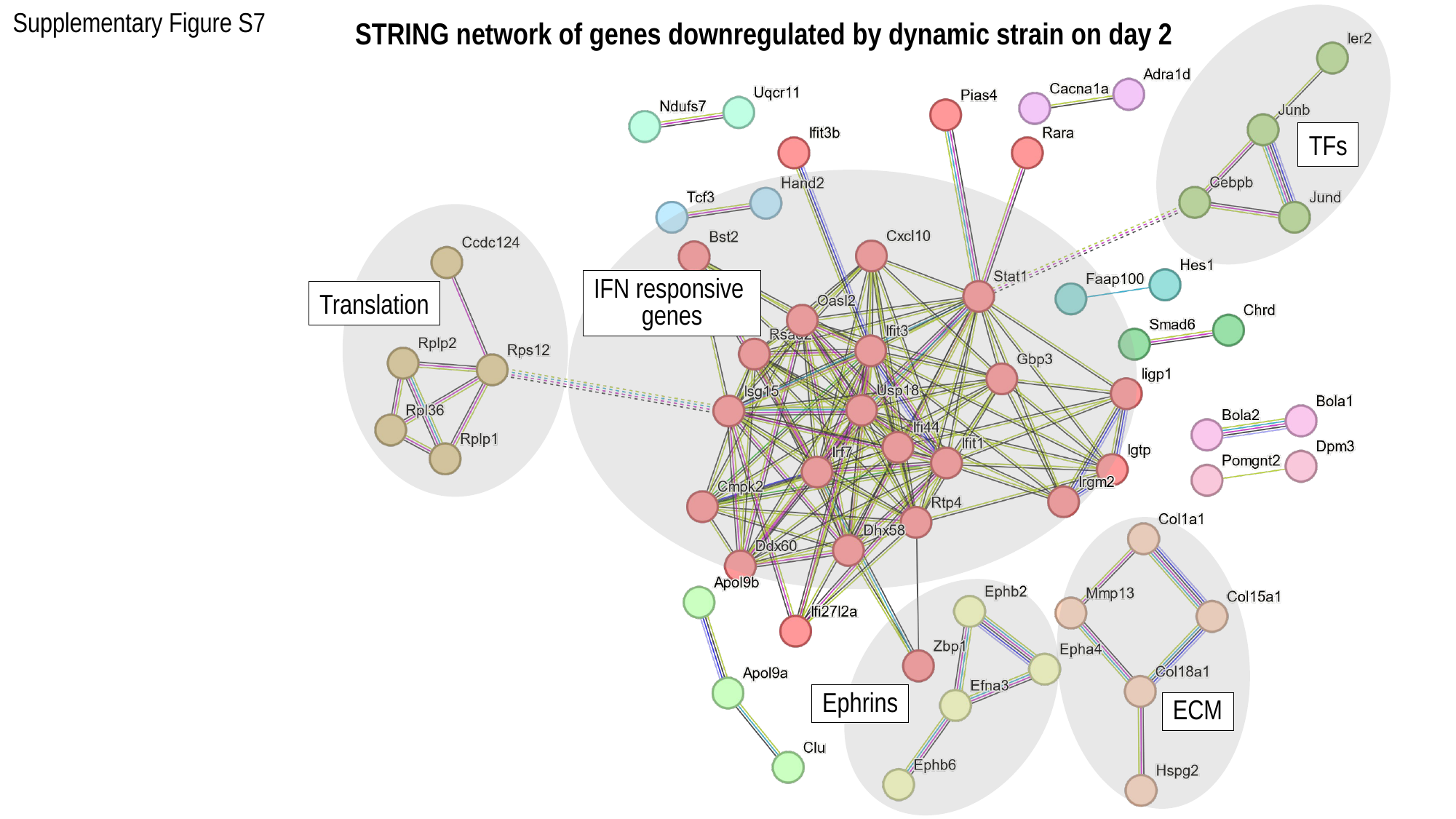

Supplementary Figure S7
STRING network of genes downregulated by dynamic strain on day 2
TFs
IFN responsive
genes
Translation
Ephrins
ECM
